# Leadership and credition: Followers' neural response to leaders who are perceived as transformational

**DOI:** 10.3389/fnbeh.2022.943896

**Published:** 2022-11-04

**Authors:** Sabine Bergner, Robert Rybnicek, Karl Koschutnig

**Affiliations:** ^1^Department of Psychology, University of Graz, Graz, Austria; ^2^Department of Corporate Leadership and Entrepreneurship, University of Graz, Graz, Austria

**Keywords:** credition, implicit leadership theories (ILTs), neuroimaging (anatomic and functional), transformational leadership (TL), neuroleadership

## Introduction

Transformational leadership (TL) has gained much attention in current leadership research (Zhao and Li, 2019) as it results in superior organizational, team and individual performance (Wang et al., 2011). According to the follower-centric leadership approach, a leader's level of transformational behavior is not only dependent on the leader's *action* but also on the follower's *perception* and *belief* (Brown, 2018). For instance, followers who believe their leader to be more transformational—irrespective of the leader's actual behavior—show higher commitment and extra effort at work (Felfe and Schyns, 2010). This suggests that TL is also in the eye of the beholder and affected by follower beliefs (Howell and Shamir, 2005).

This fMRI-study is the first to investigate the followers' neural reaction to *perceived* transformational leadership and provides novel insights into the question why TL matters. It examines the neural patterns that are activated when followers believe a leader to be transformational and examines whether these patterns relate to the level of perceived TL. Furthermore, it investigates whether followers' neural activations predict their motivation at work.

### Transformational leadership and its perception

TL describes a leadership approach that focuses on transcendent and superior goals (Antonakis and Day, 2018). At its core, it creates positive change and *transforms* followers so that they “transcend their own self-interests for the good of the group, organization, or society” (Bass, 1990, p. 53), resulting in followers doing “more than they intended and […] even thought possible” (Bass, 1998, p. 4). To induce the intended follower transformation, leaders ought to *create an attractive future vision* (inspirational motivation), *support followers* (individualized consideration), *set high ethical standards* (idealized influence) and *stimulate followers' creative thinking* (intellectual stimulation).

Previous research has illustrated several positive follower reactions to perceiving TL. For instance, followers feel more valued and optimistic, experience more positive emotions and sense higher moral values. They feel positively connected to their leader, experience more fairness and regard their work as more important (Pillai et al., 1999; Dirks and Ferrin, 2002; Turner et al., 2002; Bono and Judge, 2003; Kark et al., 2003; Keller, 2006; Bono et al., 2007; Tims et al., 2011). Importantly for this study, the *affect* tied to the followers' positive reaction when perceiving TL is seen as the most proximate follower reaction to TL (Ng, 2017) and might be a central reason why transformational leaders show impact.

### Followers' neural reaction to transformational leadership

So far, research on followers' *neural reaction* to TL is theoretical in nature. However, neuroimaging research conducted by Schjoedt et al. (2011) and Molenberghs et al. (2017) provides initial support for the assumption that leadership, in a broader sense, activates distinct neural patterns. Even though this research focused neither on TL nor on the business context, we build on it and assume that perceiving TL triggers distinct neural activations. More detailed, we expect TL to trigger the followers' *dopaminergic reward circuit*. This assumption is based on the following considerations: First, TL provokes reactions that represent well-known affective phenomena studied in social and affective neuroscience, e.g., TL triggers follower optimism, trust, generosity and fairness, all phenomena examined in neuroscience (Davidson et al., 2009); Second, according to results from social and affective neuroscience, these phenomena trigger the dopaminergic reward circuit, e.g., individuals who perceive trust, fairness and generosity activate the ventral striatum (Mobbs et al., 2009; Izuma et al., 2010; Shenhav and Greene, 2010). Additionally, those who feel optimistic display activations in the amygdala and rostral anterior cingulate cortex (Sharot et al., 2007); Third, among the diverse mechanisms underlying TL, its rewarding value is particularly important. Those who experience TL feel rewarded (Tee, 2015), a feeling which again corresponds to activation in the dopaminergic reward circuit (Liu et al., 2011). Concluding, perceiving TL is thought to trigger the dopaminergic reward circuit.

### Research focus

Based on the fact that those affective reactions reported by followers who perceive TL trigger the *dopaminergic reward circuit*, we expect that perceiving a leader to be transformational triggers the same circuit. Furthermore, we assume a positive relation between the intensity of the neural activations and the perceived level of TL, as the reward circuit activations correlate with the level of positive affect, emotion and mood (Haber and Knutson, 2010).

**Hypothesis 1**: Followers who believe their leader to be transformational—irrespective of the actual behavior—display activations in their dopaminergic reward circuit.

**Hypothesis 2**: The more followers believe their leader to be transformational, the stronger will be their neural activations.

As this is—to the best of our knowledge—the first neural study on followers' perception of TL, we also address the question of whether neuroimaging insights predict follower outcomes (Waldman et al., 2017). Therefore, we exploratively study whether the followers' neural response to perceived TL relates to their motivation at work, an outcome frequently examined in the business context.

**Research question**: Does the followers' neural response to perceived TL relate to their motivation at work?

## Method

Forty-four (29♀, *M*_age_ = 25.00, *SD*_age_ = 2.26) healthy MBA students participated in the study. They were screened for exclusion criteria (metal implants, physical impairment, pregnancy, psychosis), provided written informed consent and received a fixed compensation (€15).

In the experiment's *pre-scanning part*, participants were told a cover story to help them establish the follower role. Accordingly, they had the chance for an internship supervised by recognized leaders (both male). Depending on their task performance in the MR-scanner they would be recruited by one of two leaders—one transformational (TL), the other not (nonTL). The better their task performance in the MR-scanner, the higher their chance for being selected by the transformational leader; contrariwise the chance for the nonTL leader arose.

Both leaders and their leadership behavior were introduced using audio vignettes and portrait pictures. Participants listened to a speech that was given by each leader and saw a portrait picture of each. Importantly, study participants did not know that (a) both leaders were fictional characters (pictures obtained from Neutralized Faces Database; Ebner, 2008), (b) the speeches were derived from Kirkpatrick and Locke's (1996) vignettes on TL/nonTL and that (c) professional announcers recorded the speeches. Leaders, speeches, announcers and portrait pictures were counterbalanced and randomized.

**Figure 1:**
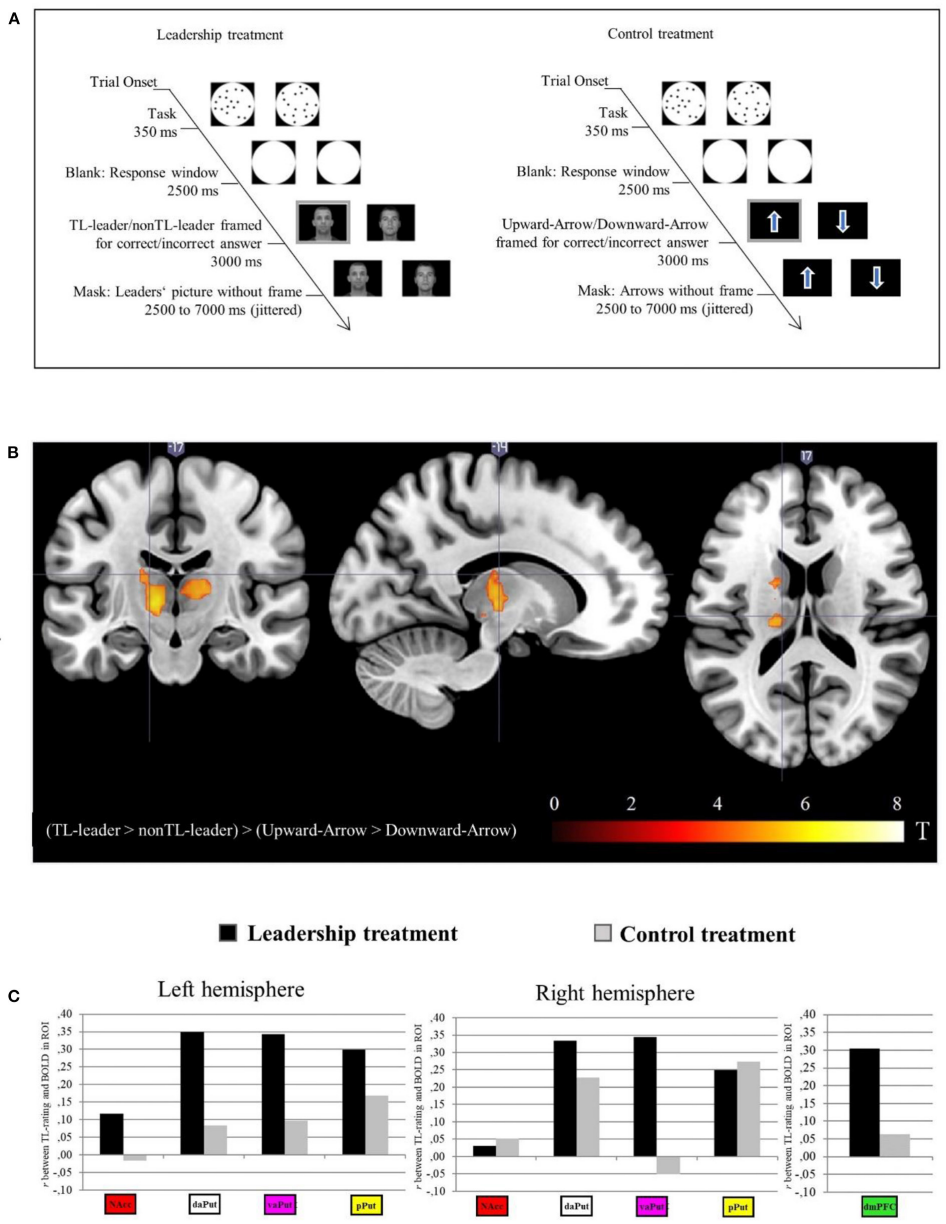
Paradigm **(A)**, whole-brain analysis **(B)** and correlation between BOLD-signal and behavioral ratings on transformational leadership **(C)**. NAcc, Nucleus accumbens; daPut, dorsal anterior putamen; vCaud, ventral caudate; vaPut, ventral anterior putamen; dCaud; dorsal caudate; pPut, posterior putamen; dmPFC, dorsomedial prefrontal cortex. Facial images reproduced with permission from the Max Planck Institute for Human Development, Center for Lifespan Psychology, Berlin, Germany, available at https://faces.mpdl.mpg.de/.

In the *scanning part*, an event-related design with a leadership and control treatment was conducted^1^. In every treatment, participants completed 50 of the trials depicted in Figure 1A. Each trial began with the performance task in which two circles with dots were displayed. Participants had to decide which of the circles held more dots (see Dehaene et al., 2005; Costa et al., 2011). If the task was solved correctly, then—in the leadership treatment—the portrait picture of the TL-leader was framed, otherwise the opposing picture was framed. In the control treatment an upward-/downward-facing arrow was framed when the task was solved correctly/incorrectly. For motivational reasons, we adjusted the task so that all participants completed 60% of the trials correctly.

In the *post-scanning part*, participants rated the two leaders' TL behavior using the Multifactor-Leadership-Questionnaire (Bass and Avolio, 1995). The likability of and motivation to work for the leaders were each assessed with a single-item scale (5-point rating).

Neural activity was measured using the blood-oxygen-level-dependent (BOLD) signal. This works by detecting the changes in blood oxygenation and blood flow that occur in response to neural activity. Before the BOLD-signal was analyzed, systematic non-task-related sources of variability were removed (e.g., artifacts due to head movement). Then, general linear modeling (GLM) was used for the first-level analyses to identify an increase/decrease of the BOLD-signal in response to the treatment or baseline signal (Dimoka, 2012; Dulebohn et al., 2016). In a third step, second-level analyses were conducted to make inferences about the whole participant group. Finally, region of interest (ROI) analysis was used to focus on the activations in predefined brain areas that are central to the reward circuitry (Kätsyri et al., 2012; see Figure 1C). Analyses were corrected for multiple comparisons. Whole brain activations were family-wise-error (FWE) corrected using a voxel-level FWE of *p* < 0.05 as a measure of significance. Additionally, mean percent signal change was extracted for each ROI using MarsBaR software (Brett et al., 2002). Additional information on the MRI procedure, data acquisition, data analysis and on the method in general is provided in the Online supplement.

## Results

Confirming the different TL-levels in the leader treatment, the TL-leader received higher TL-ratings than the nonTL-leader (*t*_(1, 42)_ = 32.55, *p* < 0.01; *M*_TL−leader_ = 4.40, *SD*_TL−leader_ = 0.34 vs. *M*_nonTL−leader_ = 1.52, *SD*_nonTL−leader_ = 0.39). Regarding hypothesis 1, the simple activation contrast of the leadership (TL-Leader > nonTL-Leader) and control contrast (Upward-Arrow > Downward-Arrow) were studied to account for activation from answering the task correctly. Table 1 and Figure 1B reveal that followers who believe their leader to be transformational activate the putamen, thalamus and supplementary motor area, which largely supports hypothesis 1.

**Table 1:** *T*-values for significantly activated voxels, MNI coordinates, and cluster sizes.

**Experimental effect**	**MNI coordinate**			**Voxels**	**Peak *T***
**Hemisphere/Region**	***x***	***y***	***Z***		
**Main effect leadership treatment (TL-leader** **>** **nonTL-leader)**					
R Caudate nucleus	18	8	−11	798	7.70
L Putamen	−21	2	−14	247	7.94
L Medial orbital frontal gyrus	3	35	−14	134	7.55
R Middle occipital gyrus	18	−103	−5	74	6.18
R Middle cingulum	0	−37	37	127	6.52
L Superior frontal gyrus	−21	32	52	41	5.90
L Cerebellum	−15	−79	−17	101	6.42
R Cerebellum	42	−70	−35	86	6.74
**Main effect control treatment (upward-arrow** **>** **downward-arrow)**					
R Caudate nucleus	9	8	−8	23	6.27
**Contrast of the simple contrasts from the leadership and control treatment**					
**(TL-leader** **>** **nonTL-leader)** **>** **(upward-arrow** **>** **downward-arrow)**					
L Putamen	−21	8	7	68	4.43
L Thalamus	−12	−16	1	103	5.35
R Thalamus	15	−19	10	62	4.63
L Supplementary motor area	−6	14	46	172	5.30

Data were corrected for multiple comparisons on a voxel-level (FWE, p < 0.05).

Regarding hypothesis 2, the BOLD response beta values for the TL-leader > nonTL-leader contrast were extracted for the predefined ROIs and correlated with the behavioral TL-ratings (MLQ-rating). Figure 1C demonstrates positive correlations between activations of the daPut (left/right), vaPut (left/right), and dCaud and TL-ratings. Importantly, the beta values of the control contrast (Upward-Arrow > Downward-Arrow) did not correlate with the TL-ratings. Thus, hypothesis 2 is largely supported.

Findings on the research question demonstrate that the followers' motivation to work for a leader significantly relates to the parameter estimates of the TL-leader > nonTL-leader in these ROIs: daPut (left *r* = 0.31, *p* < 0.05; right *r* = 0.33, *p* < 0.05), vaPut (left *r* = 0.31, *p* < 0.05; right *r* = 0.33, *p* < 0.05) and pPut (left *r* = 0.31, *p* < 0.05; right *r* = 0.39, *p* < 0.05). The activation in these ROIs explained $R_{\text{adj}}^{2}$ = 15% of the variance in follower motivation (*F*_2,41_ = 8.25, *p* < 0.01). Hierarchical regressions on follower motivation also showed that the BOLD-signal for the pPut (right) added validity over behavioral TL-ratings (Δ*R*^2^ = 0.07, *p* < 0.05; controlled for leader likability). Thus, followers' neural response to perceived TL correlates with their motivation and adds incremental validity over TL-ratings when predicting motivation.

## Discussion, conclusion and limitation

This study has two central findings. First, it reveals that the *pure belief* of a leader being transformational triggers distinct neural activations in the followers' reward circuitry. Second, it demonstrates that the neural response to perceived TL not only correlates with the followers' level of motivation but even predicts it beyond well-established rating measurements.

Regarding the first finding, this study revealed that followers who believed their leader to be transformational show activation in parts of their reward circuitry, which included the putamen, thalamus and SMA. These brain areas became even more strongly activated the more followers believed their leader to be transformational. Notably, neither personal interaction with nor actual behavior from the leader was necessary to trigger these brain areas. Therefore, the finding supports the *social construction perspective* of leadership (Keller, 2006), according to which leadership is partly constructed in the mind of followers and therefore to a certain extent independent of the actual leader behavior or leader–follower interaction.

Our results highlight the relevance of the reward circuitry when processing perceived TL. This is a novel insight and adds to findings from Schjoedt et al. (2011) and Molenberghs et al. (2017) who conducted the only existing fMRI-studies in the field but examined leadership in a rather general sense and neither focused on TL or the business context. As the reward circuitry is triggered when individuals experience rewarding or hedonistic values, it might be argued that followers feel rewarded or valued when processing TL. This assumption is supported by results showing that TL resembles an *idealized* leadership prototype which is loaded with appealing, rewarding and attractive ideas about how leaders behave (Hartog et al., 1999). As such beliefs represent so-called implicit leadership theories (Eden and Leviatan, 1975), our findings not only add a neural layer to the idea that TL represents idealized leadership but also offer insights into the neural underpinning of implicit leadership theories. Additionally, the relevance of the reward circuitry neurologically supports the well-known, yet only behaviorally examined link between TL and positive follower affect like optimism, trust or generosity (e.g., Bono and Judge, 2003; Bono et al., 2007; Bregenzer et al., 2019) as these phenomena commonly trigger the dopaminergic reward circuit (Mobbs et al., 2009; Izuma et al., 2010; Shenhav and Greene, 2010).

Regarding the second finding of interest, our results demonstrate that followers' neural responses to TL correlate with their level of motivation and even predict it beyond traditional leadership ratings. Given that this study examined the neural foundation of followers' *subjective beliefs* in a leader's TL-level, this finding highlights the relevance of *beliefs* in leadership. Therefore, it also strengthens theoretical considerations of the credition model (Seitz et al., 2018), according to which belief structures shape actions and influence motivation. While existing research supports this notion—e.g., in the educational (Mitropoulou et al., 2018) and health settings (Meissner, 2017), this study primarily validates the credition model in the business context and further offers a neural underpinning thereof.

As with any study, there are limitations. As we only investigated TL, no conclusions regarding other leadership behaviors can be drawn. Furthermore, only male leaders were examined. Therefore, it remains unclear whether female leaders would trigger similar findings. Finally, no individual differences among followers were considered. As such differences affect the perception of TL (Felfe and Schyns, 2006), future studies need to elaborate the impact of these differences. Despite these limitations we feel that our findings offer an important step toward understanding the neural mechanisms underlying leadership powers.

## Author contributions

SB was the PI and had the lead in writing the paper. KK did the analyses and co-wrote the paper. RR set up the experiment and co-wrote the paper. All authors contributed to the article and approved the submitted version.

## Funding

This paper is funded by Rüdiger Seitz, via the Volkswagen Foundation, Siemens Healthineers, and the Betz Foundation. The external funders were not involved in the study design, collection, analysis, interpretation of data, the writing of this article or the decision to submit it for publication.

## Conflict of interest

The authors declare that the research was conducted in the absence of any commercial or financial relationships that could be construed as a potential conflict of interest.

## Publisher's note

All claims expressed in this article are solely those of the authors and do not necessarily represent those of their affiliated organizations, or those of the publisher, the editors and the reviewers. Any product that may be evaluated in this article, or claim that may be made by its manufacturer, is not guaranteed or endorsed by the publisher.

## Footnotes

^1^This study was part of a multiple-study design with various treatments (see Rybnicek et al., 2019). Here we focus only on the leadership and control treatment.
