# Supplementary material for: Leadership and credition: Followers' neural response to leaders who are perceived as transformational
Source: Front Behav Neurosci. 2022 Nov 4;16:943896. doi: 10.3389/fnbeh.2022.943896 (PMC9674035; doi:10.3389/fnbeh.2022.943896)
Supplement: Supplementary file 1 [file Data_Sheet_1.docx]

**Leadership and credition:
Followers’ neural response to leaders who are perceived as transformational**

**Online supplementary material**

The experiment consisted of a pre-scanning, scanning and post-scanning phase. In the pre-scanning phase the participants were introduced to two different leaders as part of their cover story. In the scanning phase the fMRI procedure was conducted and in the post-scanning phase participants completed a set of questionnaires. Subsequently, detailed information on the pre-scanning, scanning and post-scanning phase is provided.

# Cover story as part of the pre-scanning phase

At the beginning of the pre-scanning phase participants were told a cover story to help them establish a follower role. They were told that they had the chance for an internship and that depending on their task performance in the MR-scanner they would be recruited by one of two leaders: one was transformational (TL-leader), the other was not (nonTL-leader). The better their task performance in the MR-scanner, the higher was their chance for getting selected for the internship by the transformational leader, contrariwise the chance for the nonTL-leader rose.

Bevor scanning, participants were introduced to the leaders and their way of leading employees. They listened to a meeting held by the leaders at the beginning of a new project while watching a portrait picture of the leader. Each leader held one meeting. However, study participants did not know that both leaders were fictional characters. The portrait pictures of the leaders (both male) were randomly chosen from the Neutralized Faces Database (Ebner 2008) and their speeches were derived from Kirkpatrick and Locke’s (1996) experimental study on transformational leadership. As shown by a number of previous studies which validated these speeches, one of those speeches represented a transformational leadership style where followers were respected, motivated, valued and inspired, while the other speech represented a non-transformational leadership style where followers were not approached or respected in a personal way and where the leader acted in a detached way (see, for instance, Felfe and Schyns 2006). The speeches were recorded by two professional announcers. The pairing of the announcers and leaders as well as the introduction to each leader and the pairing of the portrait pictures and leaders was randomized across participants.

# MRI-Procedures of the scanning phase

In the scanning phase of the experiment, an event-related design was conducted with a leadership treatment and a control treatment, whereby all participants had to complete both treatments (i.e. a within-subject design was used). In every treatment, participants had to complete 50 of the trials subsequently described. Treatments were presented in randomized order to all participants. After study participants had completed the 50 trials of a single treatment, the trials of the next treatment started.

A single trial began with the performance task. In this task, participants were asked to decide in which of two circles there were more dots. To this end, two circles with dots were displayed for 350 ms. Then the dots disappeared, and the white-filled circles remained empty. In the subsequent 2,500 ms time, participants had to indicate whether there were more dots in the left or right circle. Participants responded by either pressing a button for the left or the right circle.

If the task was solved correctly, the picture of the TL-leader (in the leadership treatment) and the upward arrow (in the control treatment) was framed for 3,000 ms. If the task was solved incorrectly, the opposing picture was framed for 3,000 ms. This means that in the leadership treatment the picture of the nonTL-leader was framed and in the control treatment the downward arrow was framed. If participants did not respond to the task within 2,500 ms then both pictures (e.g. TL-leader and nonTL-leader) were framed with a green frame. The framing then disappeared and the pictures remained on the screen for systematically varying periods of time (i.e. the pictures remained on the screen jittered in time with inter-trial intervals from 2,500 ms to 7,000 ms).

# MRI data acquisition in the scanning phase

In order to gather information on the participants’ brain activations, an fMRI-experiment was conducted. Therefore, participants were first asked to lie down on a platform next to the MR-scanner before their head was put into a head coil. Once the participants were instructed and placed on the platform, the platform was moved into the center of the MR-scanner. Participants were able to see outside of the scanner by looking at a mirror, which was mounted on the head coil. Outside of the scanner there was a screen on to which all visual stimuli (e.g. pictures of the leaders or arrows, circles with dots) were projected.

Once the experiment started the participants’ brain was scanned and images of the brain were recorded. Thus, the primary data were time series of images. In this study the blood-oxygen-level-dependent (BOLD) signal was used to measure neural activity. The basic idea of the BOLD signal can be summarized as follows: when a brain area is more active it consumes more oxygen and to meet this increased demand, blood flow increases to the activated area. Oxygenated blood has different magnetic characteristics from deoxygenated blood (Pauling and Coryell 1936). The BOLD signal works by detecting the changes in blood oxygenation and blood flow that occur in response to neural activity. It therefore allows conclusions to be drawn on whether a particular brain area contributes to a particular mental process. (For a more comprehensive description of the BOLD signal see Ashby 2011.)

All visual stimuli were presented using the software package Presentation 16.0 (Neurobehavioral Systems, Albany, CA, USA) and projected on a semi-translucent screen positioned approximately 1.5 m in front of the scanner. Imaging was done on a 3.0T Magnetom Skyra (Siemens Medical Systems, Erlangen, Germany) using a 32-channel head coil.

For each treatment, 276 BOLD-sensitive T2*-weighted functional images were acquired using a single-shot gradient-echo EPI pulse sequence (TR=2,400 ms, TE=30 ms, flip angle=90°, slice thickness=3.5 mm, distance factor=0%, base resolution 68×68, FOV=240 mm, 36 slices per volume). Images were recorded transversally in interleaved ascending mode and parallel to the anterior commissure–posterior commissure (AC-PC) plane. Additionally, high-resolution structural images were acquired using a T1-weighted MPRAGE sequence (TR=2530 ms, TE=2.26 ms, flip angle=9°, matrix 256×256, FOV=256 mm, voxel size=1 mm³ isotropic).

# MRI data analysis after the scanning phase

In this study MRI data analysis was conducted in four steps. The first step of the analysis comprised preprocessing activities where systematic non-task-related sources of variability were removed from the imaging data (e.g. artefacts due to head movement or high-frequency spatial noise resulting from the digitization of the imaging). Second, the time series analyses on each participant’s data were carried out (i.e. first-level analyses). As is common for neuroimaging studies, general linear modelling (GLM) was used for the first-level analyses to identify an increase or decrease of the BOLD signal in response to the leadership treatment versus the control treatment or baseline signal (Dimoka 2012; Dulebohn et al. 2016). In most neuroscientific studies a GLM analysis is performed for each voxel^[[1]](#footnote-1)^ and for each participant (Dulebohn et al. 2016). GLM analysis is appropriate when multiple predictors are used to indicate variability in a single, continuously distributed outcome variable. In this study, the outcome variable was the BOLD signal in each voxel and predictors included the pictures of each treatment with their two variations (pictures of the TL-leader and nonTL-leader, pictures of the upward and downward arrow). In a third step, second-level analyses were conducted to make inferences about the whole group of participants tested. Fourth, region of interest (ROI) analysis focused on the brain activity located in previously defined brain areas. The ROI analysis helped to identify whether activations in certain brain structures of the reward circuitry are linked to the perceived level of transformational leadership.

All analyses regarding brain activations were corrected for multiple comparisons. Whole brain activations were family-wise-error (FWE) corrected using a voxel-level FWE of *p*<.05 as a measure of significance; only activation clusters exceeding a spatial extent threshold of 49 voxels are presented. Additionally, mean per cent signal change was extracted for each ROI using MarsBaR software (Brett et al. June, 2002). Per cent signal change (describing the relative change of the BOLD signal to an individual baseline) was correlated with behavioral ratings of transformational leadership to study if participants who perceive a leader to be more transformational show more activations than those who perceive the leader as less transformational. According to Kätsyri et al. (2012), three anatomical ROIs were defined: NAcc, caudate nucleus and putamen. ROIs were defined using the Wake Forest University PickAtlas toolbox (Maldjian et al. 2003).

Functional MRI data analysis was performed using Statistical Parametric Mapping 8 (SPM8; Wellcome Dept. of Imaging Neuroscience, London, UK). Preprocessing steps included motion correction, slice time acquisition correction, and spatial normalization into the standard space (Montreal Neurological Institute; MNI) using the Echo-Planar Imaging (EPI) template provided in SPM8. Finally, the functional data were smoothed using a Gaussian filter of 8 mm. A high-pass filter with a cut-off frequency of 1/128 Hz was employed in order to remove low-frequency drifts.

At the first level of the analysis, each participant’s preprocessed data were analyzed using the principles of the general linear model (GLM), as implemented in SPM8, on a voxel-by-voxel basis. Thus, we modelled regressors within each trial (Correct vs. Incorrect, as indicated by the green framing when the task feedback was given; please see Fig. 1a). Additionally, we modelled three regressors of no-interest (Mask, Fixation and Motor Response, which indicated individual reaction time). Linear t-contrasts (Correct > Incorrect and Incorrect > Correct) were computed.

At the second level, the contrast images from all participants were entered into a one-sample t-test to assess the population mean effects. At this point it is important to note that the picture of the TL-leader was always framed when the task was solved correctly, whereas the picture of the nonTL-leader was always framed when the task was solved incorrectly. Thus, the simple contrast in the leadership treatment can be described as ‘TL-leader_correct_ > nonTL-leader_incorrect_’ or vice versa. The same holds true for the control treatment. Since the simple contrasts of the treatments are confounded with correct/incorrect answers, a contrast of the simple contrasts was calculated in order to identify brain regions sensitive to transformational leadership but controlling for activations that evoke from answering a task correctly (see e.g., Gureckis et al. 2011). Thus, the following contrast of simple contrasts was calculated: ‘TL-leader_correct_ > nonTL-leader_incorrect_’ > (Upward Arrow_correct_ > Downward Arrow_incorrect_). Since the simple contrast of the leadership treatment and the control treatment represents neural activations that relate to solving a task correctly, these reward-associated activations remain unnoted in the contrast of the simple contrasts.

# Questionnaires completed in the post-scanning phase

In the post-scanning phase of the experiment the study participants were asked to complete a set of questionnaires which included scales on transformational leadership, leader likability and the individual motivation to work for a leader.

*Transformational leadership behavior* was measured using the respective subscale of the Multifactor Leadership Questionnaire (MLQ; Bass & Avolio, 1995), the most widely used questionnaire for measuring the full-range model of leadership in general and transformational behavior in particular. Overall, the study participants rated 24 items on a 5-point frequency scale from (1) “never” to (5) “nearly always”. The following item is a sample of the respective scale: “He [The leader] communicates a compelling vision of the future”. The internal consistency for the scale was .84 in this study.

*Likability of the leader*. As leaders who are more liked might trigger different brain activations than leaders who are less liked by their followers, the likability of the leaders in this study was controlled. Therefore, a single-item scale was used and the participants had to rate the following statement on a 5-point scale ranging from (1) “not at all” to (5) “very much”: “How much do you personally like the leader you just heard”.

*Motivation to work for the leader*. The study participants indicated their motivation to work for the TL-leader and nonTL-leader. Therefore, they completed a single-item scale with the following item: “How motivated are you to work for the leader you just heard”. Participants rated the statement on a 5-point scale ranging from (1) “not at all” to (5) “very much”.

References

Ashby, F. G. (2011). Statistical analysis of fMRI data. Cambridge Mass. u.a., MIT Press.

Brett, M./Anton, J.-L./Valabregue, R./Poline, J.-B. (2002). Region of interest analysis using an SPM toolbox. Presented at the 8^th^ International Conference on Functional Mapping of Human Brain Sendai, Japan, June, 2002.

Dimoka, A. (2012). How to conduct a functional magnetic resonance (fMRI) study in social science research. MIS Quarterly 36 (3), 811–840.

Dulebohn, J. H./Davison, R. B./Lee, S. A./Conlon, D. E./McNamara, G./Sarinopoulos, I. C. (2016). Gender differences in justice evaluations. Evidence from fMRI. Journal of Applied Psychology 101 (2), 151–170. <https://doi.org/10.1037/apl0000048>.

Ebner, N. C. (2008). Age of face matters. Age-group differences in ratings of young and old faces. Behavior Research Methods 40 (1), 130–136. <https://doi.org/10.3758/BRM.40.1.130>.

Felfe, J./Schyns, B. (2006). Personality and the perception of transformational leadership. The impact of extraversion, neuroticism, personal need for structure, and occupational self-efficacy. Journal of Applied Social Psychology 36 (3), 708–739. <https://doi.org/10.1111/j.0021-9029.2006.00026.x>.

Gureckis, T. M./James, T. W./Nosofsky, R. M. (2011). Re-evaluating dissociations between implicit and explicit category learning: an event-related fMRI study. Journal of Cognitive Neuroscience 23 (7), 1697–1709. <https://doi.org/10.1162/jocn.2010.21538>.

Huettel, S. A./Song, A. W./McCarthy, G. (2009). Functional magnetic resonance imaging. 2^nd^ ed. Sunderland Mass., Sinauer.

Kätsyri, J./Hari, R./Ravaja, N./Nummenmaa, L. (2012). The opponent matters. Elevated fMRI reward responses to winning against a human versus a computer opponent during interactive video game playing. Cerebral Cortex 23 (12), 2829–2839. <https://doi.org/10.1093/cercor/bhs259>.

Kirkpatrick, S. A./Locke, E. A. (1996). Direct and indirect effects of three core charismatic leadership components on performance and attitudes. Journal of Applied Psychology 81 (1), 36–51. <https://doi.org/10.1037/0021-9010.81.1.36>.

Maldjian, J. A./Laurienti, P. J./Kraft, R. A./Burdette, J. H. (2003). An automated method for neuroanatomic and cytoarchitectonic atlas-based interrogation of fMRI data sets. Neuroimage 19 (3), 1233–1239. <https://doi.org/10.1016/S1053-8119(03)00169-1>.

Pauling, L./Coryell, C. D. (1936). The magnetic properties and structure of hemoglobin, oxyhemoglobin and carbonmonoxyhemoglobin. Proceedings of the National Academy of Sciences 22 (4), 210–216. <https://doi.org/10.1073/pnas.22.4.210>.

1. A voxel is a three-dimensional cube of brain tissue that commonly contains millions of neurons and tens of billions of synapses. The number of neurons and synapses in a voxel depends on the voxel size and the area of the brain that is imaged (Huettel et al. 2009). [↑](#footnote-ref-1)
